# Climatically suitable areas for *Hylobius abietis* and *Hylobius pales* : a global and regional analysis considering economic risks for pine production

**DOI:** 10.1002/ps.70152

**Published:** 2025-08-18

**Authors:** Jéssica Simão, Gabriel Dorotel da Silva Ferreira, Leonardo Rodrigues Barbosa, Fabiane dos Santos, George Correa Amaro, Cesar Augusto Marchioro

**Affiliations:** ^1^ Department of Agriculture, Biodiversity and Forests Federal University of Santa Catarina, Campus Curitibanos Santa Catarina Brazil; ^2^ Brazilian Agricultural Research Corporation (Embrapa Florestas) Colombo Brazil; ^3^ Plant Health Protection, Integrated Agricultural Development Company of Santa Catarina Santa Catarina Brazil; ^4^ Brazilian Agricultural Research Corporation (Embrapa Roraima) Boa Vista Brazil; ^5^ Postgraduate Program in Natural and Agricultural Ecossystems Federal University of Santa Catarina, Campus Curitibanos Santa Catarina Brazil

**Keywords:** ecological niche modeling, forest pests, quarantine pests, MaxEnt, normalized concentration index

## Abstract

**BACKGROUND:**

The weevils *Hylobius abietis* and *Hylobius pales* are major pests of pine species within their native ranges. Their potential spread to *Pinus*‐producing regions outside these areas could result in substantial economic and ecological losses, underscoring the need for studies that identify regions at greater risk of invasion.

**RESULTS:**

Climate suitability maps, generated using the MaxEnt machine learning algorithm, identified climatically suitable areas for *H. abietis* and *H. pales* in multiple regions beyond their native ranges. These areas include several *Pinus‐*producing countries in the Southern Hemisphere (e.g*.*, Australia, Brazil, Chile, New Zealand, South Africa), as well as locations near ports, airports, or major international trade hubs. In Brazil, 68.1% of *Pinus*‐producing areas fall within the suitable range for *H. abietis*, while 91.5% of cultivated *Pinus* areas are suitable range for *H. pales*. Among the 588 *Pinus‐*producing municipalities, 48 depend on this economic activity to a moderate or high degree. Additionally, the analysis revealed that 42.0% of these municipalities face a moderate to high economic risk in the event of an invasion by *H. abietis*, while 90.5% are at risk from *H. pales*.

**CONCLUSION:**

Our results highlight the importance of incorporating region‐specific data to improve invasion risk map accuracy and guide preventive actions against two major forest pests. Most *Pinus*‐producing municipalities in Brazil face moderate to high economic risk from potential invasions by *H. abietis* and *H. pales*, emphasizing the need for proactive prevention strategies. © 2025 The Author(s). *Pest Management Science* published by John Wiley & Sons Ltd on behalf of Society of Chemical Industry.

## Graphical abstract

Climate suitability maps indicate a high economic risk for *Pinus*‐producing municipalities in Brazil if *Hylobius abietis* and *Hylobius pales* invade, highlighting the need for preventive measures.

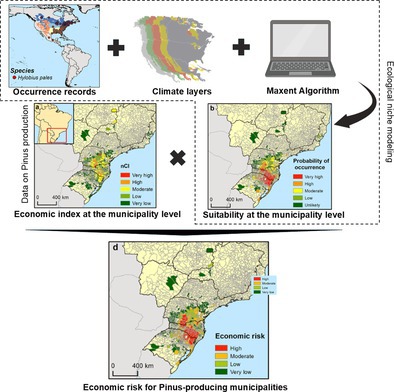

## 1 INTRODUCTION

Biological invasion is a leading cause of biodiversity loss, posing significant risks to ecosystem function, public health, and agricultural and forestry productivity.^1^, ^2^ The spread of exotic species has accelerated biotic homogenization on a global scale,^3^ and many invasive species are vectors for emerging diseases^4^ or agricultural pests affecting widely cultivated crops.^5^, ^6^ Estimates suggest that invasive species have contributed to 60% of recorded global extinctions.^7^ Likewise, the economic impact of biological invasions has quadrupled each decade, reaching an estimated global cost of US$ 423 billion in 2019.^7^

Given the invasive species' extensive environmental and socio‐economic impacts, international efforts have aimed to curb biological invasions. However, invasion rates continue to rise across most taxa,^8^ largely driven by the intensification of global trade in the 20th century.^8^ Insects, in particular, are experiencing a notable increase in new invasion records, causing an estimated economic loss of US$70 billion per year, of which approximately US$14 billion are linked to forest pests.^9^ In this context, international plant protection organizations have prioritized preventive measures. In this effort, identifying areas at high risk for the introduction of invasive species and regions most vulnerable to invasion is crucial for effective prevention.^10^ Global invasion risk maps offer a preliminary analysis of a species' potential to establish in new areas. These analyses can be further refined for insect pests using regional data on the economic importance of affected plant species to understand the potential economic impacts of an invasion.

The large pine weevil, *Hylobius abietis* (L., 1758), and the pales weevil, *Hylobius pales* (Herbst, 1797) (Coleoptera: Curculionidae) are significant pests of conifers species in their respective native range in Europe and North America, respectively.^11^, ^12^, ^13^, ^14^, ^15^ Both species are major forestry pests, particularly in young coniferous reforestation areas. Adult weevils feed on the bark of young seedlings, leading to substantial growth reductions, trunk deformation, and high mortality during the early stages of plant development.^16^, ^17^ Without protective measures, mortality rates can reach 60–80% in plants attacked by *H. abietis* ^16^ and 30–60% by *H. pales*.^18^ In Europe, *H. abietis* causes annual damage of almost €120 million.^19^

Both *H. abietis* and *H. pales* are considered polyphagous, feeding on a broad range of conifer species, including those belonging to the genus *Pinus*, one of the most widely distributed in the Northern Hemisphere.^20^ In the 20th century, several *Pinus* species were introduced to the Southern Hemisphere for forestry, reforestation, and erosion control purposes.^20^ The widespread presence of *Pinus* may facilitate the spread of insects that use these trees as hosts, such as *H. abietis* and *H. pales*. In recognition of this risk, the Regional Plant Protection Organization in South America (Comité de Sanidad Vegetal del Cono Sur) designated *H. abietis* and *H. pales* as absent quarantine species in 2018.

Ecological niche models are widely used to estimate suitable areas for insect pests, helping to identify regions at higher risk of invasion.^10^, ^21^ This approach enables the identification of climatically suitable areas on a global scale, while regional data can be incorporated to refine these projections. In this study, the MaxEnt algorithm was applied to estimate climatically suitable areas for *H. abietis* and *H. pales* worldwide. This information was then combined with data on *Pinus* distribution and economic indices related to wood production in Brazil, to prioritize areas for implementing preventive measures. The analysis also identifies municipalities that are economically most vulnerable in the event of a biological invasion followed by the insect's dispersal into suitable areas.

## 2 MATERIAL AND METHODS

### 2.1 Occurrence records and predictor variables

**Figure 1:**
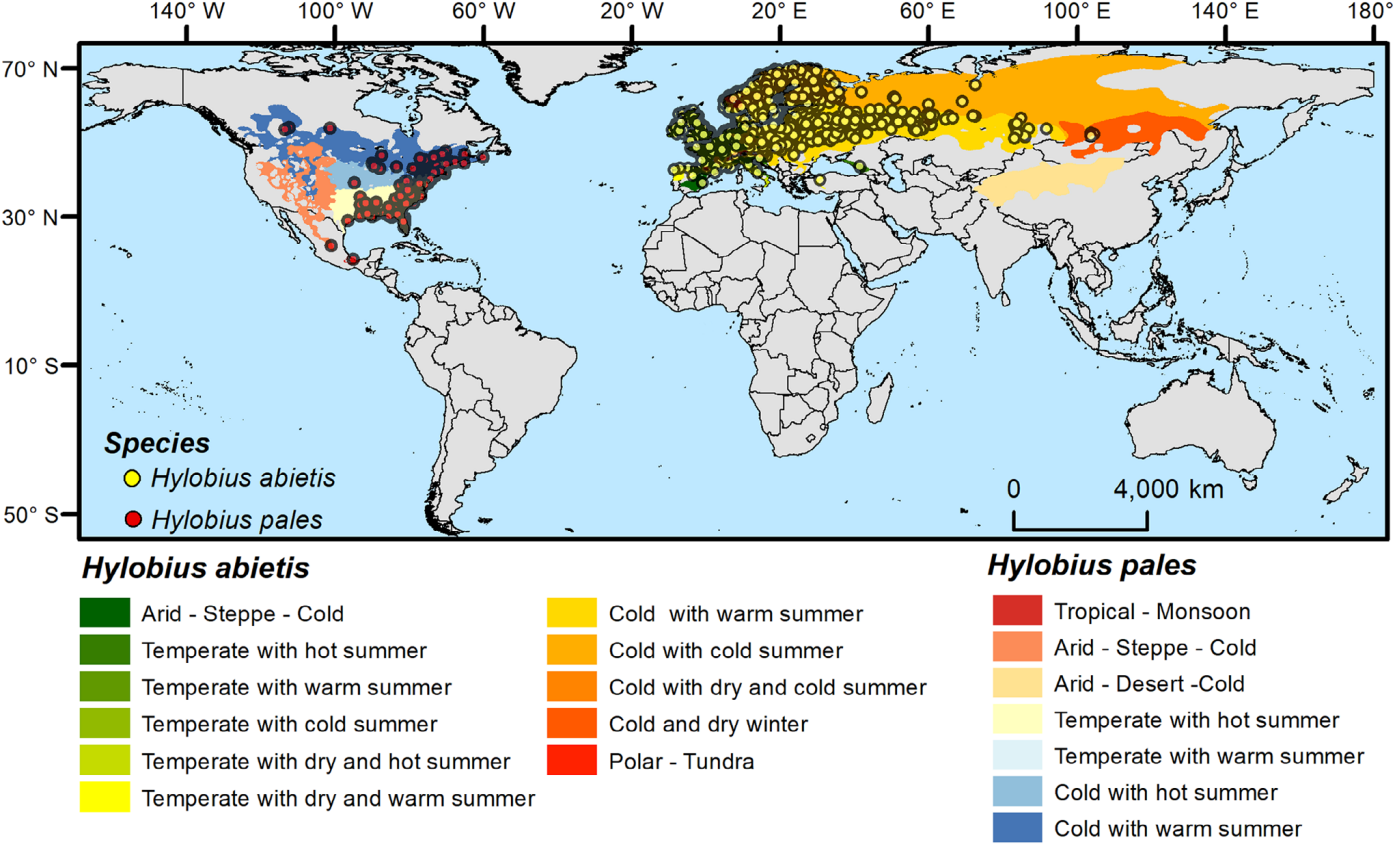
Occurrence records of *Hylobius abietis* and *Hylobius pales* obtained from the Global Biodiversity Information Facility, along with Köppen–Geiger climate zones used as background areas for each species during model training.

Occurrence records for *H. abietis* ^22^ and *H. pales* ^23^ were gathered from published literature and the Global Biodiversity Information Facility (GBIF, available at www.gbif.org) database (Fig. 1). Information on the known distribution of these species was sourced from the European and Mediterranean Plant Protection Organization (EPPO) for *H. abietis* and the Centre for Agriculture and Bioscience International (CABI) for *H. pales*, and records outside their established range were excluded from analyses. To reduce spatial autocorrelation and improve model performance,^23^ occurrence records were spatially filtered by applying a minimum distance of 20 km between records using the *spThin* package^25^ in the R statistical environment.^26^ This procedure reduced the dataset from 3183 to 2051 records for *H. abietis* and from 140 to 113 records for *H. pales*.

Elevation and 14 among the 19 bioclimatic variables from the WorldClim database (http://worldclim.org), at a 2.5 arc‐minute resolution, were used to develop the models: annual mean temperature (Bio1 – °C), mean diurnal range (Bio2 – °C), temperature seasonality (Bio4 – standard deviation × 100), maximum temperature of warmest month (Bio5 – °C), minimum temperature of coldest month (Bio6 – °C), temperature annual range (Bio7 – °C), mean temperature of wettest quarter (Bio8 – °C), mean temperature of driest quarter (Bio9 – °C), annual precipitation (Bio12 – mm), precipitation of wettest month (Bio13 – mm), precipitation of driest month (Bio14 – mm), precipitation seasonality (Bio15 – coefficient of variation), precipitation of warmest quarter (Bio18 – mm), and precipitation of coldest quarter (Bio19 – mm).^27^ These variables were chosen due to their influence on insect occurrence and abundance.^28^

### 2.2 Model development

The potential distribution of *H. abietis* and *H. pales* was predicted using the MaxEnt machine learning algorithm.^29^ MaxEnt is known for its strong statistical performance relative to other algorithms and is widely used to predict species distribution when only presence records are available.^30^, ^31^ In addition to presence records, MaxEnt uses background points randomly sampled from the study area to characterize the available environment for the species. The delimitation of this background is a key factor influencing model performance. Here, the background area for each species was defined using the Köppen–Geiger climate zones, with zones containing one or more occurrences selected as the background area (Fig. 1).^32^

Including correlated predictor variables can lead to overly complex models that are not suitable for spatial projection.^33^ To avoid this, environmental variables were first cropped using the background area as a mask. Then, correlated variables were excluded using the Variation Inflation Factor (VIF) with a threshold of 10. Annual mean temperature and annual precipitation were retained due to their relevance to insect distribution and ease of interpretation. This filtering was conducted with the *usdm* package in R, and only the remaining variables were used for model development.

Model complexity is also influenced by the functions used to transform covariates (feature classes) and the choice of regularization values. To identify the optimal combination of feature classes and regularization multiplier values for each species, 50 models were generated using the ENMeval 2.0 package.^34^ Ten regularization values were tested, ranging from 0.5 to 5 in increments of 0.5, along with five feature class combinations: L (linear), Q (quadratic), LQ (linear‐quadratic), LQH (linear‐quadratic‐hinge), and LQHP (linear‐quadratic‐hinge‐product).^35^ The best model was selected based on the corrected Akaike Information Criterion (AICc), which accounts for both model fit and complexity, with a low AICc value indicating better model performance.

The performance of the selected model for each species was evaluated using the area under the curve (AUC), which ranges from 0 to 1, with values close to 1.0 considered optimal. To test the significance of the obtained AUC, a null model approach was employed.^36^ According to this approach, AUC values were generated for 100 models with the same settings as the empirical model, but occurrence records were randomly distributed within the study area. For the AUC value of the empirical model to be considered significant, it had to exceed at least 95% of the AUC values from the simulated models. Additionally, the Continuous Boyce Index (CBI) was used, ranging from −1 to 1, with values near to 1 indicating good model quality.^37^

### 2.3 Spatial analysis

#### 2.3.1 Invasion risk

The map displaying the introduction risk (figure 1(b) in the paper of Early *et al*.^38^) based on data about airport and port presence, passenger flows, and global trade, was utilized to delineate areas with the highest probability of invasion. Initially, the original introduction risk map was transformed into values ranging from 0 (low probability) to 1 (high probability). Then, to compute the invasion risk, the suitability map generated by MaxEnt for the studied species was multiplied by the invasion probability map.

#### 2.3.2 Economic risk analysis

The value of pine production for each Brazilian municipality in 2022 was obtained from the Brazilian Institute of Geography and Statistics (IBGE) using the *datazoom.amazonia* R package.^39^ To assess the significance of pine production in each municipality, we applied the normalized Concentration Index (nCI).^40^ A detailed description of the calculation methodology can be found in Crocco *et al*.^40^ and Amaro *et al*.^41^, ^42^ Briefly, the nCI is based on three different indices used to evaluate (i) the specificity of the pine production for the municipality, (ii) the weight of pine for the municipal production, and (iii) the importance of the municipal production for the national production of pine.

The Location Quotient (LQ) was used to identify patterns of concentration or spatial dispersion, providing a measure of the specificity of a product within a municipality. A higher LQ indicates a greater specialization of the municipality in pine production.^40^ The LQ was calculated as follows:

$${\mathrm{LQ}}_{\mathrm{ij}}=\left({\mathrm{VP}}_{\mathrm{ij}}/{\mathrm{VP}}_{j}\right)/\left({\mathrm{VP}}_{\mathrm{iBR}}/{\mathrm{VP}}_{\mathrm{BR}}\right)$$

where: LQ_ij_ is the locational quotient of product i in municipality j, VPij is the value of production of product i in municipality j, VP_j_ is the value of total forestry production in municipality j, VP_iBR_ is the total production value of product i in Brazil, and VP_BR_ is the total value of forestry production in Brazil.

The second index is the modified Hirschman–Herfindahl index (HHI), used to measure the contribution of *Pinus* production in the overall forestry production of a municipality.^40^ A positive value indicates that the production of i is more concentrated in municipality j:

$${\mathrm{HHI}}_{\mathrm{ij}}=\left({\mathrm{VP}}_{\mathrm{ij}}/{\mathrm{VP}}_{\mathrm{iBR}}\right)-\left({\mathrm{VP}}_{j}/{\mathrm{VP}}_{\mathrm{BR}}\right)$$

Finally, the Relative Participation (RP) index was used to evaluate the importance of the pine production in a municipality relative to the total Brazilian production. This index was calculated using the following formula:

$${\mathrm{RP}}_{\mathrm{ij}}={\mathrm{VP}}_{\mathrm{ij}}/{\mathrm{VP}}_{\mathrm{iBR}}$$

The nCI was calculated by the sum of these three indices multiplied by their respective weights (θ), determined through a principal component analysis (PCA) based on eigenvalues and eigenvectors (for a detailed methodology, see Crocco *et al*.^40^ and Amaro *et al*.^41^, ^42^):

$${\mathrm{nCI}}_{\mathrm{ij}}=\theta_{1}{\mathrm{LQ}}_{\mathrm{ij}}+\theta_{2}{\mathrm{RP}}_{\mathrm{ij}}+\theta_{3}{\mathrm{HHI}}_{\mathrm{ij}}$$

The resulting economic index was combined with climate suitability data for *H. abietis* and *H. pales* to estimate the economic risk of a potential invasion. Initially, the probability of occurrence was calculated for each Brazilian municipality based on the average suitability within its boundaries. A two‐dimensional risk matrix was then applied to estimate economic risk, considering five classes of probability of occurrence and five nCI classes, ranging from 1 (unlikely invasion/very low importance of pine) to 5 (high probability of invasion/high pine importance), as defined by the Jenks classification method. The final economic risk was determined by averaging the classes of probability of occurrence class and nCI for each municipality. This value was then used to classify economic risk into four levels: very low (0–1.5), low (1.5–2.0), moderate (2.0–3.0), and high (3.0–5.0). All these analyses were carried out in the R environment.

### 2.4 Production area at risk

Maps displaying suitability scores from 0 to 1, generated by MaxEnt, were classified into four categories using the Jenks natural breaks method: unsuitable, marginally suitable, moderately suitable, and highly suitable for the species. Data on pine plantation areas were obtained in shapefile format from the Global Forest Watch database (www.globalforestwatch.org), based on satellite imagery with plantation boundaries manually delineated. This map was converted to a 30‐second resolution raster and overlaid onto the reclassified suitability maps to estimate the proportion of the pine plantation areas falling within zones considered moderately to highly suitable for the occurrence of *H. abietis* and *H. pales*.

## 3 RESULTS

### 3.1 Model assessment

**Figure 2:**
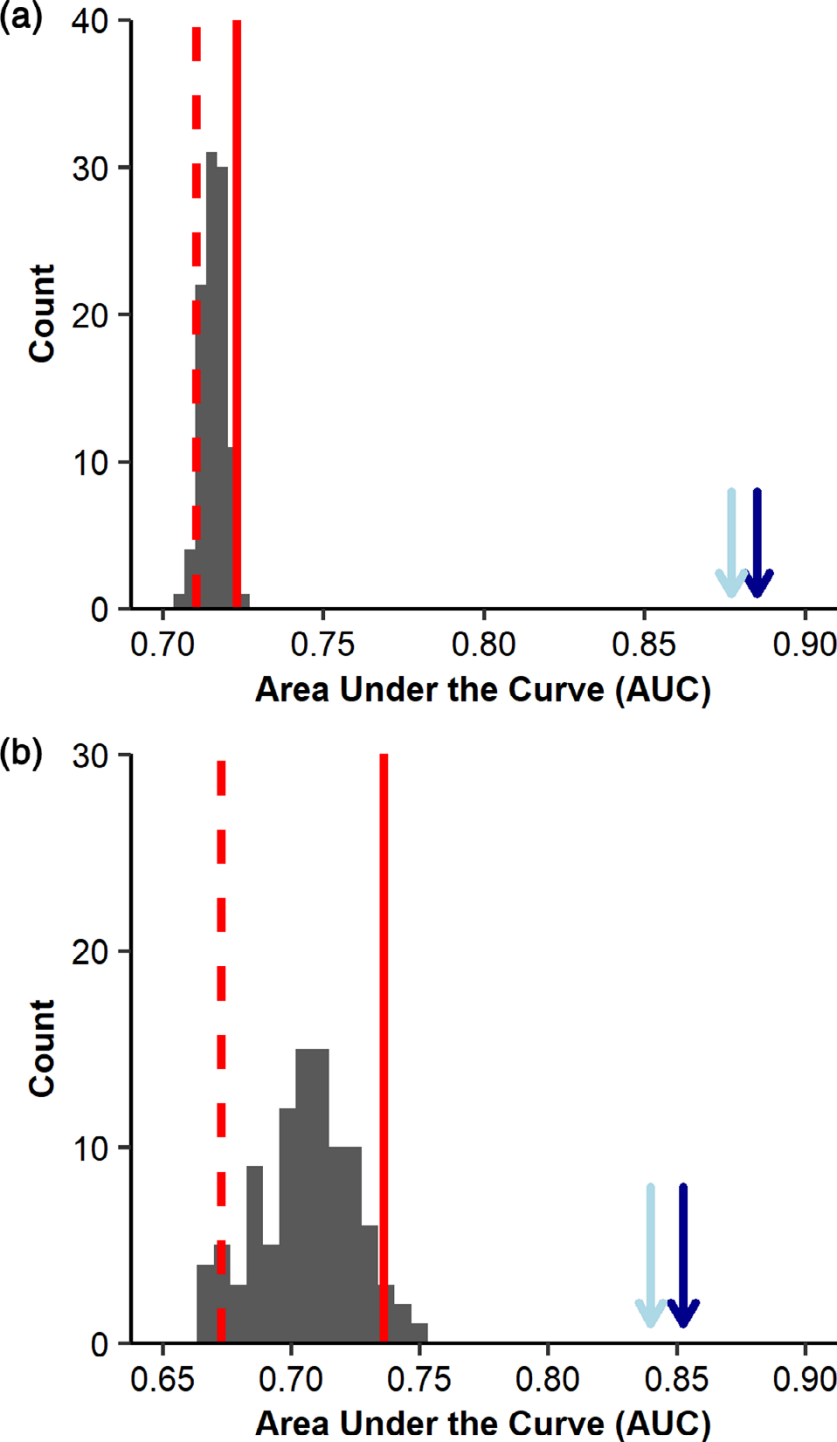
Empirical training (dark blue) and testing (light blue) area under the curve (AUC) of the receiver operating characteristic (ROC) curve for *Hylobius abietis* (a) and *Hylobius pales* (b), compared to simulated based on the null‐model approach proposed by ter Stege and Raes.^36^

The model with the optimal configuration for *H. abietis*, based on the AICc, combined linear, quadratic, hinge, and product feature classes with a regularization multiplier value of 1.0 (LQHP‐1.0). For *H. pales*, the best model included linear and quadratic feature classes with a regularization multiplier of 2.0 (LQ‐2.0). Both models demonstrated strong discriminatory ability, with AUC greater than 0.80 for *H. abietis* (AUC_TRAIN_ = 0.885; AUC_TEST_ = 0.877) and *H. pales* (AUC_TRAIN_ = 0.853; AUC_TEST_ = 0.840). The empirical AUC values were significantly higher than those obtained from the null model approach for both species (Fig. 2). The high performance of the selected models is further confirmed by the CBI values greater than 0.90 for *H. abietis* (CBI_TRAIN_ = 0.992, CBI_TEST_ = 0.929) and 0.70 for *H. pales* (CBI_TRAIN_ = 0.965, CBI_TEST_ = 0.777).

### 3.2 Variable importance

**Figure 3:**
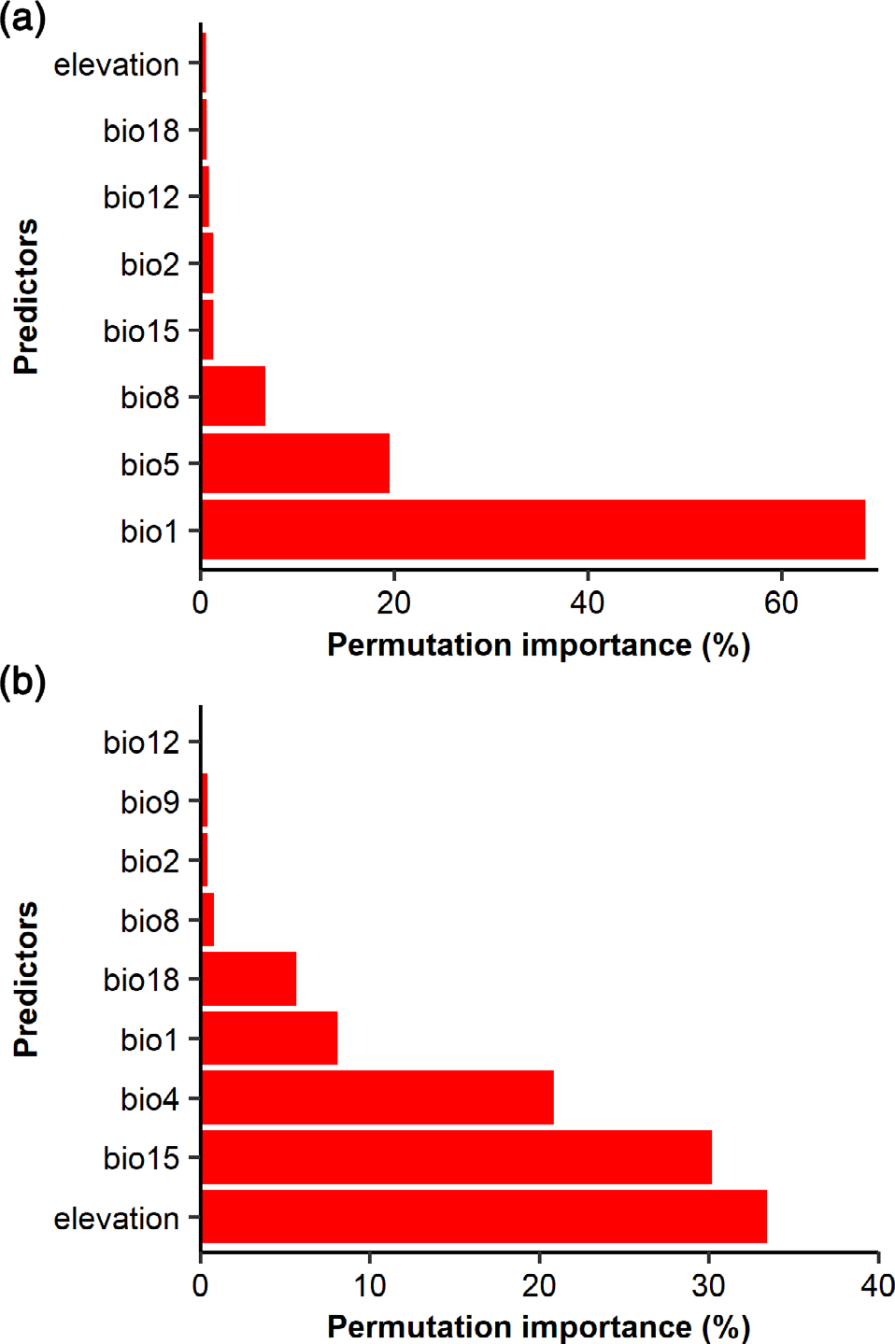
Permutation importance of the variables used in the models developed for *Hylobius abietis* (a) and *Hylobius pales* (b). Predictors are: bio1 – annual mean temperature (°C), bio2 – mean diurnal range (°C), bio4 – temperature seasonality (standard deviation × 100), bio8 – mean temperature of wettest quarter (°C), bio9 – mean temperature of driest quarter (°C), bio12 – annual precipitation (mm), bio15 – precipitation seasonality (coefficient of variation), and bio18 – precipitation of warmer quarter (mm).

Annual mean temperature (Bio1; 68.6%), maximum temperature of warmest month (Bio5; 19.6%), and maximum temperature of wettest quarter (Bio8; 6.7%) were the primary contributors to the model developed for *H. abietis*, accounting for 94.9% of the total contribution (Fig. 3). The response curves suggest that *H. abietis* prefers milder climates, as indicated by higher climatic suitability when the annual mean temperature exceeds 5 °C, and a decline in suitability with increasing maximum temperatures in the warmest month and wettest quarter (Supporting Information Fig. S1). These results are consistent with the species' native distribution, which is concentrated in regions with humid continental, temperate oceanic, and subarctic climates in its native range (Fig. S2).

For *H. pales*, the most influential variables were elevation (33.4%), precipitation seasonality (Bio15; 30.2%), temperature seasonality (Bio4; 20.8%), and annual mean temperature (8.1%), collectively contributing 92.6% to the model. The response curves showed that the suitability decreases in high‐altitude areas and regions experiencing high‐temperature seasonality (Fig. S3). According to our analysis, within its native range, this species predominantly occurs in regions with humid subtropical and humid continental climates (Fig. S4).

### 3.3 Global suitability and invasion risk

**Figure 4:**
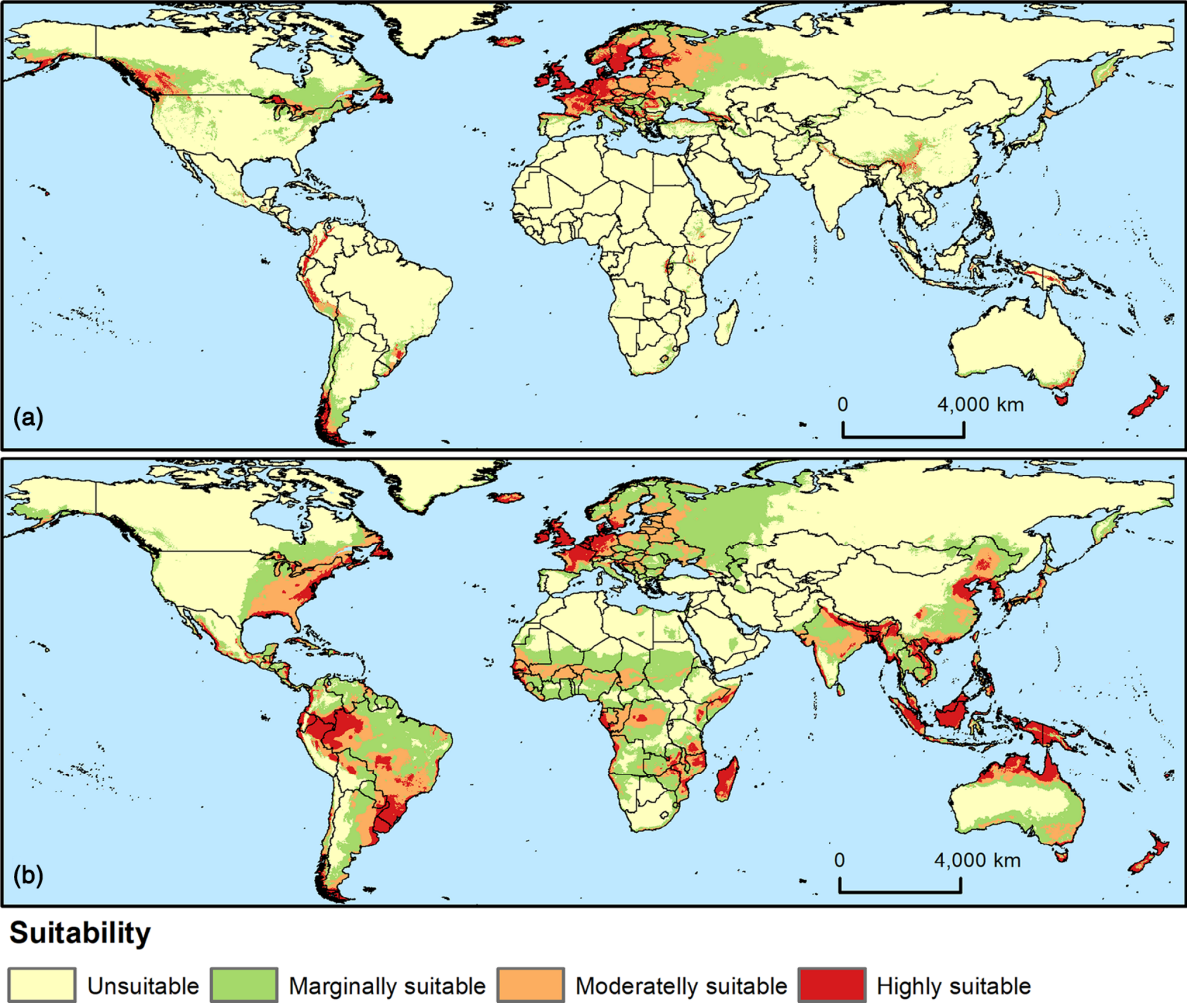
Climate suitability maps for *Hylobius abietis* (a) and *Hylobius pales* (b), generated using the MaxEnt machine learning algorithm.

Moderately to highly climatically suitable areas for *H. abietis* outside its native range were identified in North, Central, and South America; isolated regions of sub‐Saharan Africa; eastern, northern, and south‐eastern Asia; south‐eastern Australia; and New Zealand (Fig. 4). For *H. pales*, much of Central and South America was estimated to be moderately to highly suitable, along with Mediterranean, western, and northern European countries, as well as several areas in sub‐Saharan Africa, most of southeast Asia, isolated areas in south Asia, northern, southern and south‐eastern Australia, New Zealand (Fig. 4).

**Figure 5:**
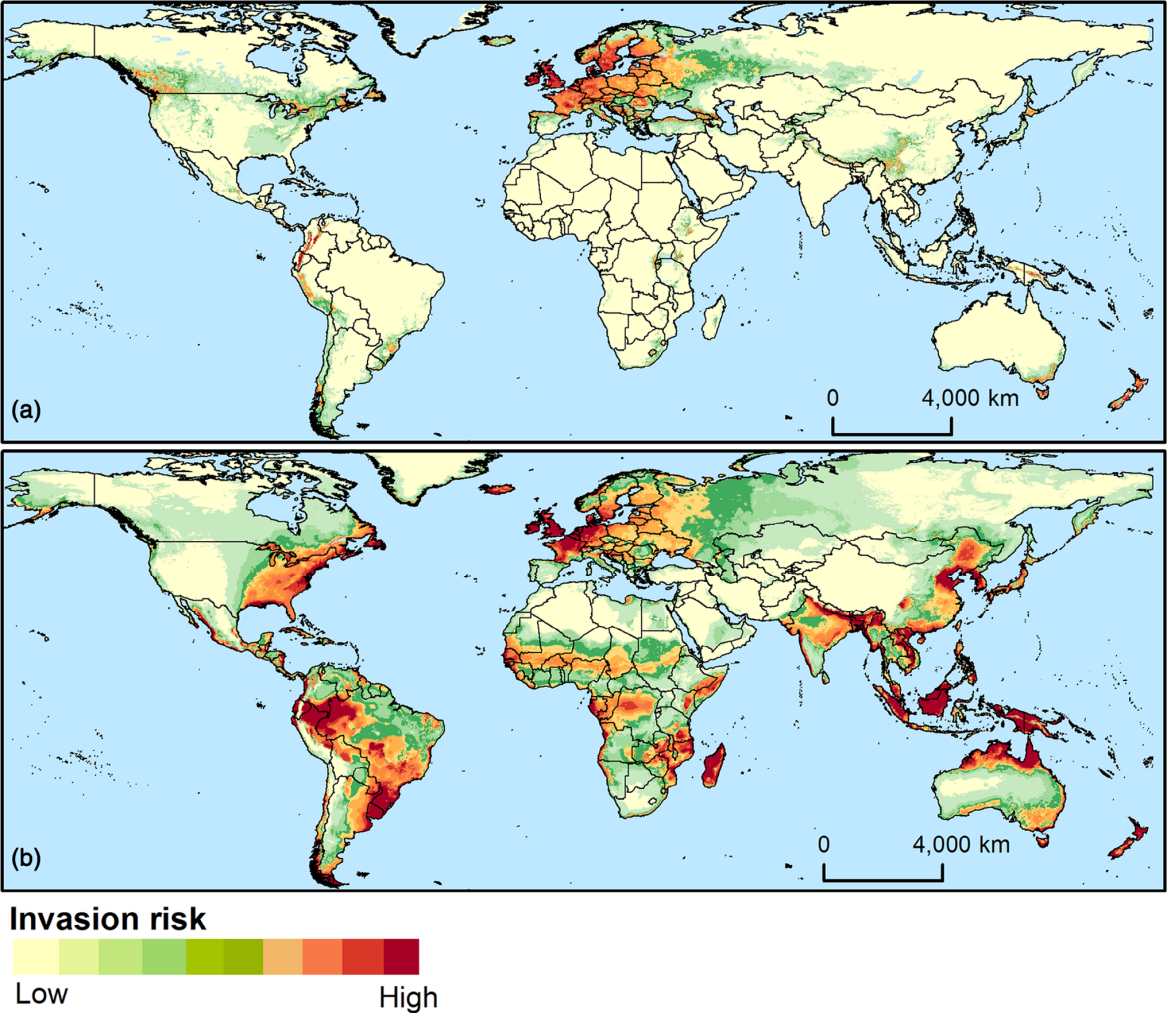
Invasion risk maps for *Hylobius abietis* (a) and *Hylobius pales* (b), derived from the combination of climate suitability and introduction likelihood maps.^38^

When climate suitability was combined with information on the introduction likelihood, high‐risk invasion areas for *H. abietis* were identified in isolated regions of south‐eastern and eastern Canada, Central and South America, including southern and south‐eastern Brazil, sub‐Saharan Africa, northern and south‐eastern Asia (particularly China), south‐eastern Australia, and most of New Zealand (Fig. 5). Since a larger territorial extent was predicted as highly suitable for *H. pales*, its invasion risk is also higher. For instance, several regions in Central and South America, western and northern Europe, southeast Asia, Australia, and New Zealand were identified as high‐risk areas of invasion (Fig. 5).

### 3.4 Economic risk

**Fig. 6:**
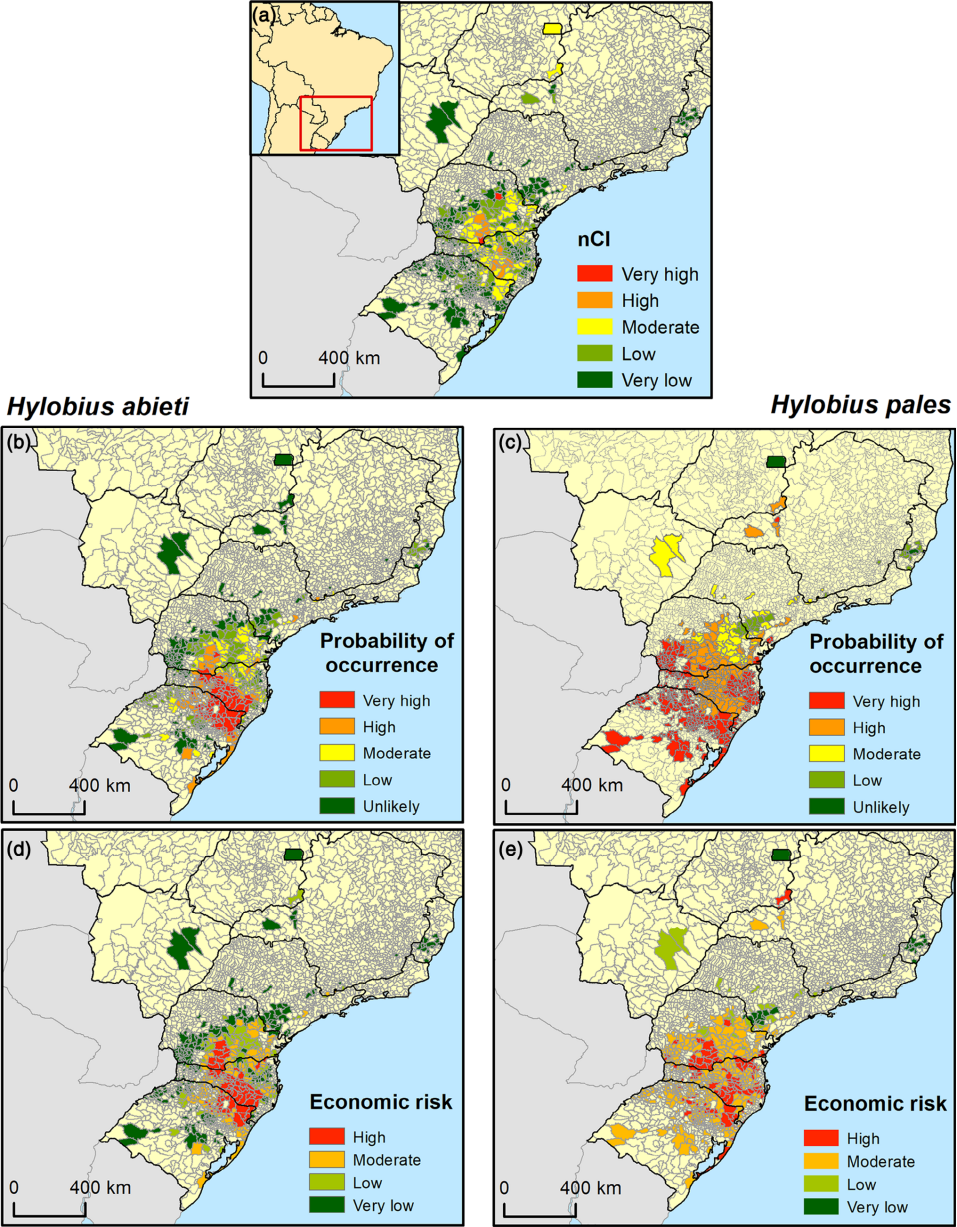
Economic risk maps for *Hylobius abietis* (d) and *Hylobius pales* (e) across *Pinus*‐producing municipalities in Brazil. The economic risk maps were generated by combining an economic index representing the importance of *Pinus* production in each municipality (a) with the climate suitability for *H. abietis* (b) and *H. pales* (c).

The nCI effectively captured the variation in the importance of *Pinus* production across Brazilian municipalities (Fig. 6(a)). Most *Pinus* production is concentrated in southern Brazil, specifically in the states of Paraná (23.6%), Santa Catarina (29.8%), and Rio Grande do Sul (36.9%). Among the 588 *Pinus‐*producing municipalities, 48 depend on this economic activity to a moderate to very high degree. The majority of these municipalities are in Santa Catarina (47.9%), followed by Paraná (41.7%) and Rio Grande do Sul (6.3%).

When combined with climate suitability, our analysis revealed that 28.7% (169) and 13.3% (78) of *Pinus*‐producing municipalities face moderate and high risk of invasion by *H. abietis*, respectively (Fig. 6(d)). The situation is even more concerning for *H. pales*, with 70.9% (417) and 19.6% (115) of municipalities at moderate and high risk, respectively (Fig. 6(e)). For both species, the municipalities at the highest risk of invasion are primarily in southern Brazil (Fig. 6(d),(e)). Most of the *Pinus*‐producing municipalities identified at high‐risk for *H. abietis* and *H. pales* are in Santa Catarina, representing 56.4% and 49.1% of cases, respectively. However, a significant proportion of *Pinus*‐producing municipalities in Paraná and Rio Grande do Sul are also at risk, totaling 10.3% and 33.3% for *H. abietis* and 23.7% and 27.2% for *H. pales*, respectively.

### 3.5 Pinus‐producing areas at risk

The extent of overlap between *Pinus*‐producing areas and suitable regions for *H. abietis* and *H. pales* varied by species. For *H. abietis*, 68.1% of *Pinus*‐producing areas fall within its suitable range, with 29.5% in marginally, 24.8% in moderately, and 13.8% in highly suitable zones. The situation is even more concerning for *H. pales*, as all cultivated *Pinus* areas in Brazil lie within its suitable range, including 1.6% in marginally, 21.14% in moderately, and 67.2% in highly suitable zones (Fig. S5).

## 4 DISCUSSION

Identifying areas at risk of invasion by invasive species is crucial for developing effective preventive measures.^2^, ^21^ In this context, suitability maps generated by ecological niche models provide an initial indication of where a species might establish if introduced. The planning of preventive measures can be enhanced by incorporating regional information on potential entry points and host plant availability. In this study, we integrated climate suitability for two important pine pests, *H. abietis* and *H. pale*, with information on the spatial distribution and economic importance of pine in Brazil. The analysis revealed that a significant portion of *Pinus*‐producing areas falls within the suitable range of both species Additionally, the economic assessment highlighted that these species pose a substantial threat to the economy of several municipalities in Brazil.

The reliability of the projections depends on the ability of the models to accurately predict species distributions. The statistical metrics used to assess model performance indicate that they effectively distinguished between suitable and unsuitable habitats for *H. abietis* and *H. pales*. Additionally, the models successfully predicted the native distribution of both species. The response curves further suggest that the models captured key ecological aspects of the species. For instance, annual mean temperature and maximum temperature of the warmest month were among the most influential variables for *H. abietis*. Habitat suitability increases with temperature up to approximately 20 °C. This pattern aligns with the occurrence of *H. abietis* predominantly in humid continental, temperate oceanic, and subarctic climates. In these climate zones, the annual average temperature remains below 22 °C, with at least 4 months exceeding 10 °C.^43^ Similarly, precipitation seasonality emerged as a key predictor in the model for *H. pales*. This species primarily inhabits humid subtropical and humid continental climates in the United States and Canada, which are characterized by relatively even rainfall distribution throughout the year.^43^ The response curve generated for *H. pales* indicates that habitat suitability decreases as precipitation seasonality increases.

Several regions beyond the native range of *H. abietis* and *H. pales* have been identified as suitable habitats. Many of these areas are located near ports, airports, or major international trade hubs, increasing the likelihood of introduction. Additionally, numerous *Pinus* species were introduced to the Southern Hemisphere during the 20th century for forestry, ornamental planting, reforestation, and erosion control.^20^ Following their introduction, many *Pinus* species spread into natural ecosystems,^44^, ^45^ and today, suitable host plants for the weevils are present across multiple countries in the Southern Hemisphere. In Brazil, for instance, *Pinus taeda* L. and *Pinus elliotti* Engelm. were initially restricted to forestry areas but later expanded into adjacent regions.^46^ This spread was likely driven by ecological traits that facilitate their dispersal, establishment, and rapid growth.^47^, ^48^

The presence of large‐scale plantations with high densities of a single species or genus, combined with inadequate management practices, increases the risk of pest establishment in the event of an invasion.^49^ In addition to the expansion of extensive monocultures, certain silvicultural practices have inadvertently created conditions favorable to the development of *H. abietis* and *H. pales*, including the presence of stumps from tree cutting, high densities of trunks of the same age, and seedling plantations in clear‐cut areas.^50^ Such conditions are observed in several countries where *Pinus* has been introduced, including Brazil.

The economic damage caused by *H. abietis* and *H. pales* in their native ranges is an additional concern for Pine‐producing countries within the species' climatically suitable range. *Hylobius abietis* is considered a major pest of conifers in Europe,^11^, ^14^, ^15^, ^51^ primarily in young coniferous reforestations.^50^ Damage is highly variable depending on the location and cultural practices. In southern and central Sweden, reforestations planted with unprotected conifer seedlings recorded an average of 30% plant mortality,^52^ while in Northern Ireland newly planted seedlings mortality was 90%.^53^ A similar situation is observed with *H. pales* in North America east of the Great Plains and north of Ontario, where adults attack young conifer seedlings causing economic losses of 30–60% in the first year.^18^ If *Pinus* species outside the weevils' native range serve as hosts, severe economic losses could occur in the event of an invasion.

The vast areas of *Pinus* plantations, the long history of forest pest invasions worldwide, and the economic losses they have caused in their native range justify concerns about the potential spread of *H. abietis* and *H. pales* to new regions. In this context, prioritizing areas based on regional characteristics is essential for generating risk maps that support the development of preventive measures. Following the methodology proposed by Amaro *et al*.,^42^ this study employed a two‐dimensional risk matrix to create risk maps, assessing and prioritizing risks according to their likelihood of occurrence and potential impact. The likelihood of occurrence was derived from ecological niche models, while the potential impact was estimated using an economic index (nCI) representing the significance of *Pinus* production in each municipality. A key advantage of this approach is its ability to generate invasion risk data at the municipal level, an administrative scale that facilitates the implementation of preventive measures by phytosanitary agencies. Additionally, recognizing the potential economic losses associated with an invasion of these species can help anticipate social challenges in municipalities economically dependent on *Pinus* cultivation. However, this analysis relies on crop production databases, which may not always be available in all regions.

The nCI revealed that 8.3% of *Pinus‐*producing municipalities depend on *Pinus* production as a key economic activity, with most of these municipalities located in southern Brazil. This region provides a climatically suitable environment for both species and, consequently, 38.6% and 88.3% of *Pinus‐*producing municipalities were identified as being at moderate to high risk in the event of an invasion by *H. abietis* and *H. pales*, respectively. Additionally, *H. abietis* is known for its high migratory potential and strong long‐distance dispersal ability. During migration periods, mature adults of this species can travel up to 80 km, although most individuals typically fly distances of up to 10 km.^54^, ^55^ In the case of invasion, this species could rapidly spread to the areas identified as suitable, potentially causing severe economic losses.

Despite their high dispersal capacity, the availability of climatically suitable areas, and the presence of host plants in various regions of the world, the potential for the introduction of *H. abietis* and *H. pales* can be considered low. The widespread distribution of *H. abietis* in Europe is believed to have resulted from the transport of wood containing larvae and pupae and, less frequently, adults.^56^ However, according to CABI,^52^ it is unlikely that this species would be introduced into another country through loose wood packaging material, processed or treated wood, non‐timber materials, solid wood without bark or debarked wood. In this context, introduction to distant countries is more likely to occur via the transport of seedlings, as larvae lodge near the root collar region.^52^ Alternatively, although less probable, introduction could occur through bark‐covered wood, which may serve as shelter for adults. Nevertheless, studies assessing the areas at highest risk of invasion remain relevant, as the damage caused by invasive species outweighs the cost of prevention.

Although ENM packages are a widely used tool to assess the potential distributions of invasive species, their predictions should be interpreted with caution due to inherent uncertainties of the correlative approach employed. A key limitation lies in the assumption of niche conservatism, despite growing evidence that some species may undergo niche shifts during biological invasions.^57^ In such cases, ENM packages may underestimate the species' potential distribution range. To address this limitation, suitability maps can be updated by incorporating new occurrence records as species expand into regions with non‐analogous climates.^58^ Another potential limitation is the use of a single algorithm to generate suitability maps. While some authors advocate for consensus maps derived from multiple algorithms, arguing that they offer greater reliability,^59^ this approach is not consistently supported by empirical studies designed to test that hypothesis.^60^, ^61^ In the present study, we employed an algorithm recognized for its strong predictive performance and followed the most recent methodological recommendations in the literature regarding mode development and evaluation.^24^, ^30^, ^33^, ^35^

## 5 CONCLUSION

In conclusion, this study generated global risk maps for the invasion of *H. abietis* and *H. pales* based on climate suitability and the likelihood of introduction. The analysis revealed that several regions beyond the species' native range face a high risk of invasion. Additionally, these maps were refined with regional‐scale data from Brazil, incorporating information on *Pinus* plantations and their economic importance at the municipal level. The results indicate that a significant portion of *Pinus*‐producing municipalities are at moderate to high risk, highlighting the potential for severe economic losses in the event of an invasion by *H. abietis* and *H. pales*. Our findings underscore the importance of integrating regional data to enhance invasion risk maps and provide valuable insights for developing preventive measures against these two significant forest insect pests.

## Supporting information


**Figure S1.** Response curves for each predictor included in the model developed for *Hylobius abietis*.
**Figure S2.** Density of *Hylobius abietis* occurrence records across Köppen–Geiger climate classification zones.
**Figure S3.** Response curves for each predictor included in the model developed for *Hylobius pales*.
**Figure S4.** Density of *Hylobius pales* occurrence records across Köppen–Geiger climate classification zones.
**Figure S5.** Climate suitability maps for *Hylobius abietis* (a) and *Hylobius pales* (b) predicted using the MaxEnt machine learning algorithm, alongside Pinus‐producing areas in Brazil.

## Data Availability

The data that support the findings of this study are available from the corresponding author upon reasonable request.
