# Supplementary material for: Climatically suitable areas for Hylobius abietis and Hylobius pales : a global and regional analysis considering economic risks for pine production
Source: Pest Manag Sci. 2025 Aug 18;81(12):8477–86. doi: 10.1002/ps.70152 (PMC12618907; doi:10.1002/ps.70152)
Supplement: Supplementary file 1 — Figure S1. Response curves for each predictor included in the model developed for Hylobius abietis. Figure S2. Density of Hylobius abietis occurrence records across Köppen–Geiger climate classification zones. Figure S3. Response curves for each predictor included in the model developed for Hylobius pales. Figure S4. Density of Hylobius pales occurrence records across Köppen–Geiger climate classification zones. Figure S5. Climate suitability maps for Hylobius abietis (a) and Hylobius pales (b) predicted using the MaxEnt machine learning algorithm, alongside Pinus‐producing areas in Brazil. [file PS-81-8477-s001.pdf]

APPENDIX

Climatically suitable areas for *Hylobius abietis* and *H. pales*: a global and regional analysis considering economic risks for pine production

Jéssica Simão, Gabriel Dorotel da Silva Ferreira, Leonardo Rodrigues Barbosa, Fabiane dos Santos, George Correa Amaro, Cesar Augusto Marchioro

Response curves

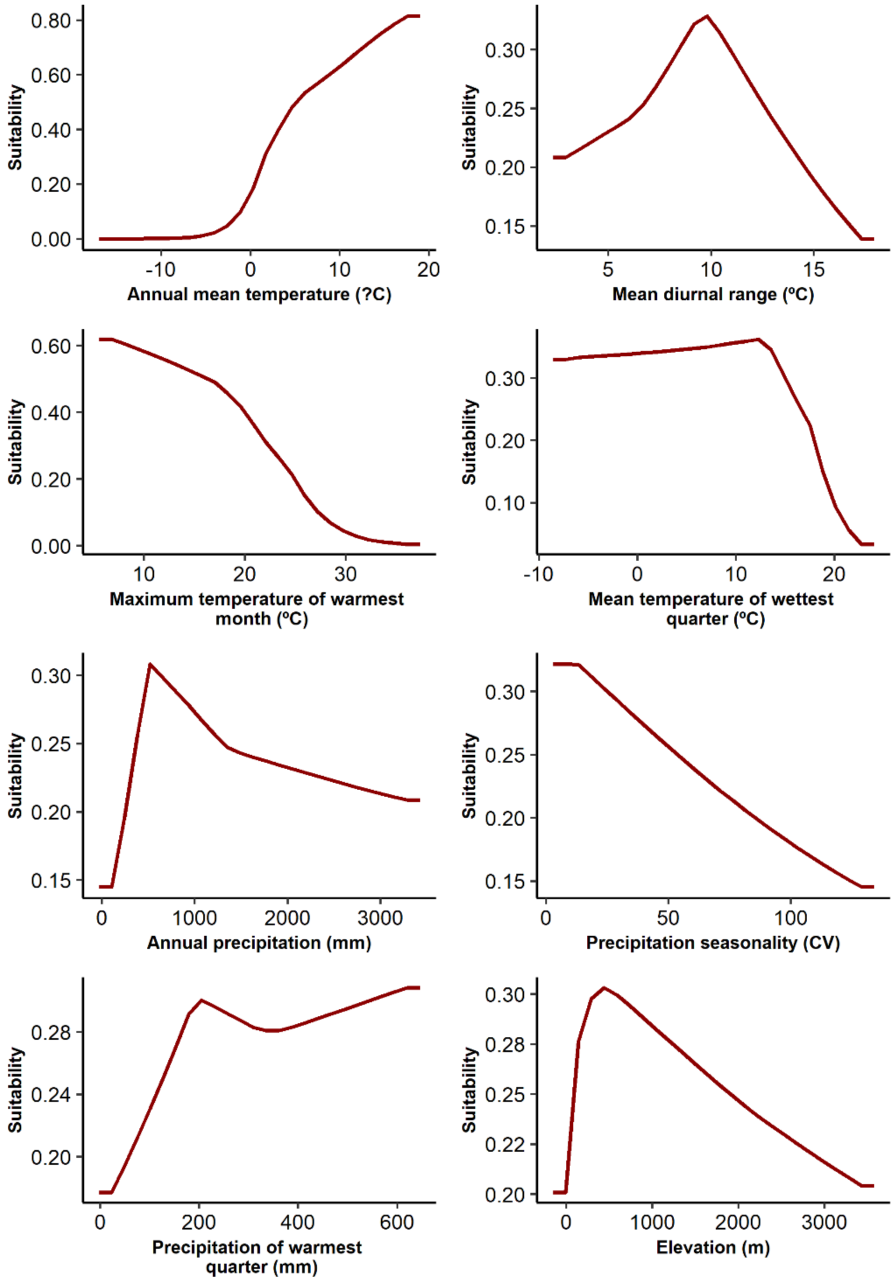

Figure S1 Response curves for each predictor included in the model developed for *Hylobius abietis*.

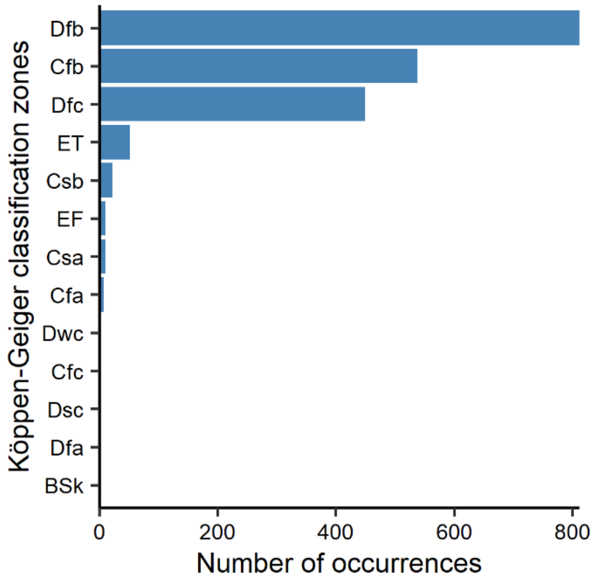

Figure S2 Density of *Hylobius abietis* occurrence records across Köppen-Geiger climate classification zones.

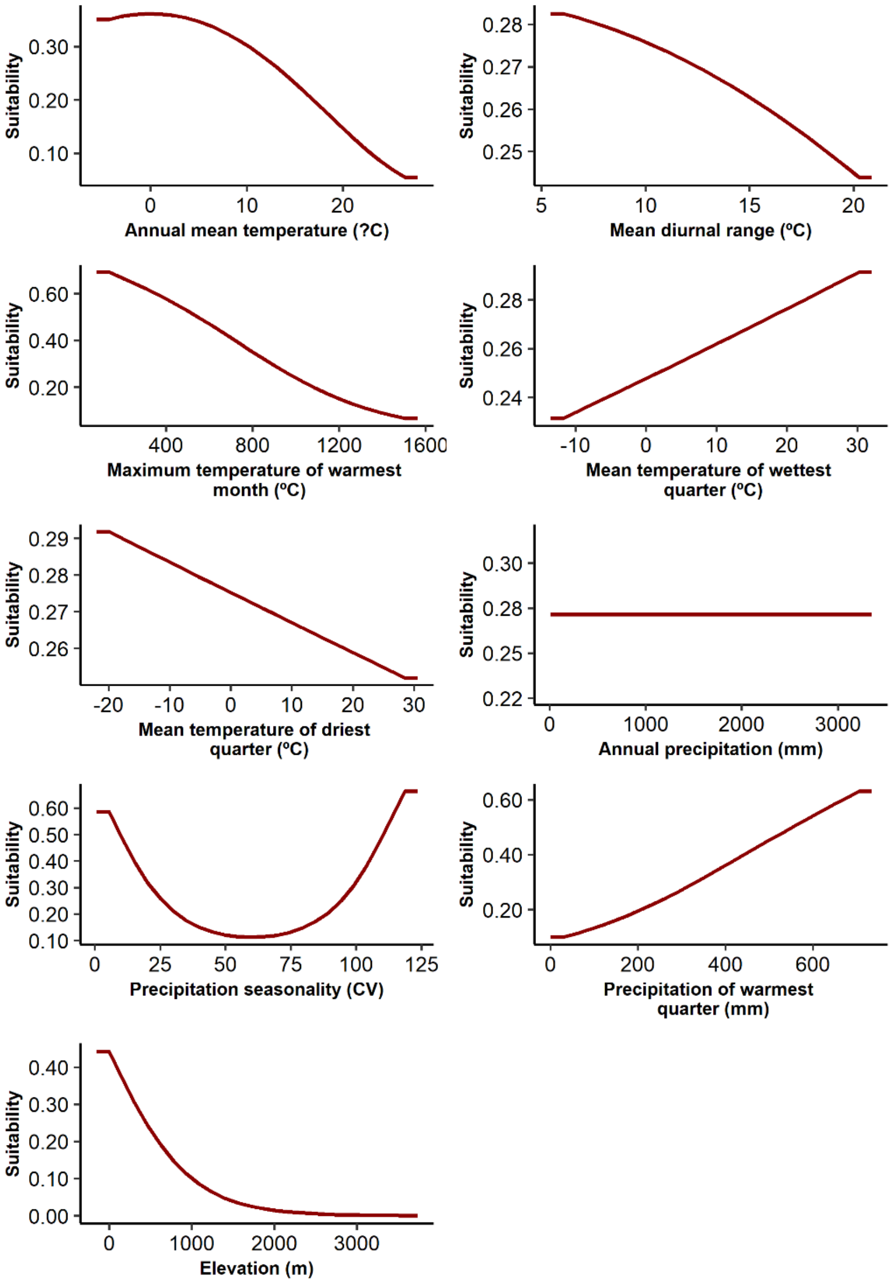

Figure S3 Response curves for each predictor included in the model developed for *Hylobius pales*.

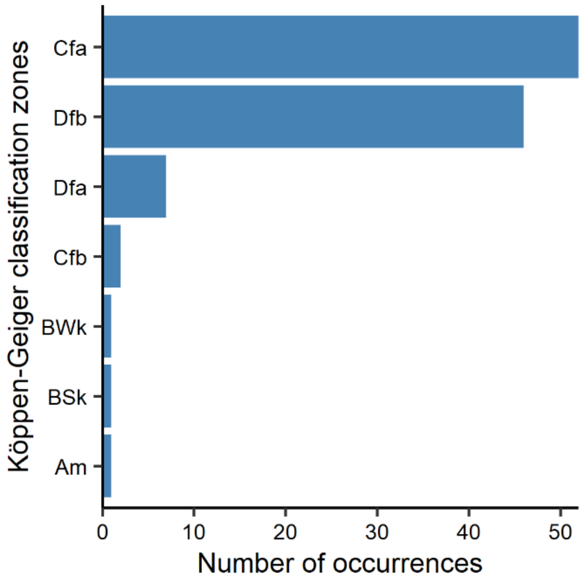

Figure S4 Density of *Hylobius pales* occurrence records across Köppen-Geiger climate classification zones.

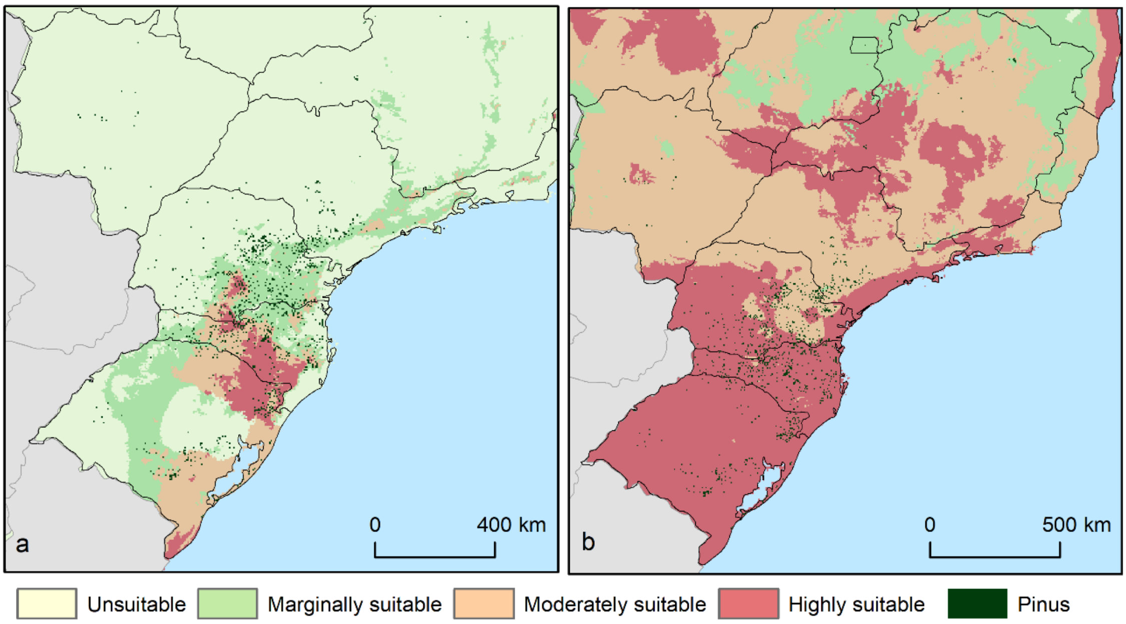

Figure S5 Climate suitability maps for *Hylobius abietis* (a) and *H. pales* (b) predicted using the MaxEnt machine learning algorithm, alongside Pinus-producing areas in Brazil.
